# Genetic utility of natural history museum specimens: endangered fairy shrimp (Branchiopoda, Anostraca)

**DOI:** 10.3897/zookeys.457.6822

**Published:** 2014-11-25

**Authors:** Adam R. Wall, Daniel Campo, Regina Wetzer

**Affiliations:** 1 Natural History Museum of Los Angeles County, 900 Exposition Boulevard, Los Angeles, CA 90007 USA; 2 University of Southern California, Molecular and Computational Biology, Los Angeles, CA 90089 USA

**Keywords:** Museum specimens, Anostraca, Branchinectidae, *Branchinecta sandiegonensis*, *Branchinecta lynchi*, *Branchinecta lindahli*, endangered, threatened, vernal pool, California

## Abstract

We examined the potential utility of museum specimens as a source for genetic analysis of fairy shrimp. Because of loss of their vernal pool habitat, some fairy shrimp (including *Branchinecta sandiegonensis* and *Branchinecta lynchi*) are listed as threatened or endangered in Southern California by the United States Fish and Wildlife Service. Management of those species requires extensive population genetics studies and the resolution of important genetic complexity (*e.g.* possible hybridization between endangered and non-endangered species). Regulations mandating deposition of specimens of listed species have resulted in thousands of specimens accessioned into the Natural History Museum of Los Angeles County that have been preserved in a variety of solutions. We subsampled those specimens, as well as other Anostraca with known collection and preservation histories, to test their potential for genetic analysis by attempting DNA extraction and amplification for mt16SrDNA. Fixation and preservation in not denatured ethanol had a far greater sequencing success rate than other (and unknown) fixatives and preservatives. To maximize scientific value we recommend field preservation in 95% not denatured ethanol (or, if pure ethanol is unavailable, high-proof drinking spirits, *e.g.* Everclear™, or 151 proof white rum), followed by storage in 95% not denatured ethanol.

## Introduction

The largest collection of endangered Southern Californian fairy shrimp in the United States of America is at the Natural History Museum of Los Angeles County (LACM). The LACM is working closely with the United States Fish and Wildlife Service (USFWS) to increase the scientific value of these specimens for both morphological and molecular studies. Fairy shrimp occur in ephemeral vernal pool habitats worldwide (Keeley and Zedler 1998). In densely human populated areas, their fragile habitats continue to be severely degraded and many have been destroyed by urbanization (Bauder and McMillan 1998, King 1998, Simovich et al. 2013).

At least 15 plant species are recognized as threatened or endangered in California vernal pool habitats, but only a few invertebrates are similarly recognized (USFWS 2005). *Branchinecta conservatio*, *Branchinecta longiantenna*, and *Branchinecta sandiegonensis* are listed as “Endangered”, and *Branchinecta lynchi* is listed as “Threatened” by the USFWS. In California, the USFWS issues permits for the collection of fairy shrimp and requires the deposition of endangered and threatened species in one of two repositories: the LACM or the California Academy of Sciences in San Francisco. Traditionally, Southern California specimens come to the LACM and northern California collections go to the California Academy. Since 1995 about 5,000 lots of *Branchinecta lindahli*, *Branchinecta lynchi*, and *Branchinecta sandiegonensis* have been accessioned into the LACM collections. This represents about 95% of our total anostracan holdings.

Simovich et al. (2013) suggest that human disturbance is increasing the generalist *Branchinecta lindahli*’s range, which in turn is eroding the native range of *Branchinecta sandiegonensis*. Due to increasing sympatric distribution of these species, these authors (and Fugate 1998 before them) claim that the endangered and non-endangered species (*Branchinecta sandiegonensis* and *Branchinecta lindahli*) are hybridizing, thereby threatening the genetic integrity and persistence of *Branchinecta sandiegonensis*. Using a PCR-based screen using mitochondrial DNA to determine maternal lineage, in conjunction with morphological examination, Simovich et al. (2013) claim putative hybrids share their maternal DNA with the more common species at a site. Unfortunately, their claims are not testable or reproducible as the specimens used in their study are unavailable. Aside from this study, only an unpublished master’s thesis exists that addresses genetic aspects of putatively hybrid populations of Southern California *Branchinecta sandiegonensis* (Andrews 2013). That study depended on prior researchers’ assessments of hybridization in individual pools. These claims of hybridization underscore the need for comprehensive molecular studies to characterize the actual genetic diversity and species boundaries of Southern California fairy shrimp before further management and remediation recommendations are made.

In contrast to the lack of work being conducted on endangered Southern Californian fairy shrimp, there has been a large amount of work studying the genetics and phylogeographics of the endangered Californian salamander *Ambystoma tigrinum* (Amphibia: Caudata: Ambystomatidae) (Ryan et al. 2009, Johnson et al. 2010, Johnson et al. 2011). These studies were made possible in large part by a very extensive collection of samples — tail clippings — of *Ambystoma tigrinum* that span the salamander’s geographic range through the last 25 years. Just as important as the breadth of the collection of tail clippings was that these samples were preserved with a method that made them accessible for molecular study decades later. The findings from these studies have already helped the management of *Ambystoma tigrinum* by identifying which populations have the greatest genetic diversity and allowing USFWS to target high value populations for increased protection (Johnson et al. 2011). The LACM is working closely with USFWS to assemble a collection of endangered Californian fairy shrimp necessary for similar genetic and phylogeographic studies. Both the LACM and USFWS fully expect that one day such studies will help better inform and shape the management of endangered fairy shrimp.

In this study we test whether preservation in pure not denatured ethanol makes anostracan museum specimens more readily accessible for molecular studies over anostracan museum specimens that had historically been fixed in denatured ethanol, isopropyl, or even acetone, then transferred into pure not denatured ethanol. Our study compares the success rates of amplifying a fragment of mt16SrDNA for specimens preserved in not denatured ethanol and for specimens in other preservatives. Because of their rarity and the difficulty in collecting fresh fairy shrimp specimens, being able to use specimens already in museum collections would be advantageous. To improve the utility of future collections, we suggest improvements in field and post-field preservation and handling based on our findings. If adopted, these improvements will greatly enhance the genetic usefulness of specimens and thereby allow more thorough assessments.

## Methods

### Material examined

We first inventoried, digitized, and georeferenced our entire anostracan collection — approximately 5,000 lots. We selected 50 specimens from across the taxonomic range that had been contributed by different collectors and consulting companies using a range of different field preservatives prior to deposition at the LACM (at the LACM, all specimens are transferred from the field preservative into fresh museum-grade not denatured ethanol). We then attempted to amplify a ~550 bp mt16SrDNA fragment (see Table 1).

**Table 1.** Extractions and amplifications attempted for this publication. Taxa arranged in alphabethical order. Locality, specimen collection date, collector, and preservative are as transcribed from specimen labels. Specimen condition and body part used in extraction are indicated if this information was recorded. Double-stranded DNA concentration in ng/µL. Qubit value indicated as low, *i.e.*, 0<0.05 ng/µL. Asterisk (*) indicates sequence was generated and is listed in Table 2.

	Taxon	Date of collection	Description of preservative on label	Locality	Collector	Part of specimen used	Extraction number	Outcome	dsDNA ng/µL 0<0.05
1	*Artemia monica*	06-Jul-90	70% ethanol	California, Mono County, Mono Lake, south Tufa Reserve	H. Kuck	1 broken specimen	2013	contaminated; blasts as *Homo*	0
2	*Artemia monica*	01-Jan-10	fixed and preserved in 95% ethanol	California, Mono County, Mono Lake	M. Hauser	1 whole squished specimen	2008	*beautiful sequence	39.1
3	*Branchinecta coloradensis*	23-Apr-92	70% ethanol	California, Lassen County, Hog Flat Reservoir	King, Gluesenkamp, Tritt	1 broken specimen	2003	failed	0
4	*Branchinecta dissimilis*	23-Mar-92	70% ethanol	California, Shasta County, Fall River	King, Gluesenkamp, Kloock	2 broken pieces	2017	failed	0.2
5	*Branchinecta gigas*	unknown	acetone	California, San Bernadino County, Mojave Desert	J. Martin, J. Plum	2 phyllopods only	1990	failed	0.17
6	*Branchinecta gigas*	unknown	not indicated	Washington, Grant County	unknown	1 small whole specimen	2006	failed	0.13
7	*Branchinecta gigas*	unknown	not indicated	Washington, Grant County	unknown	dissected off egg sack with eggs	2007	failed	0
8	*Branchinecta lindahli*	27-Dec-12	fixed and preserved in 95% ethanol	California, San Diego County, Marine Corps Base Camp Pendleton	L. Woolley	not recorded	2036	failed	no data
9	*Branchinecta lindahli*	27-Dec-12	fixed and preserved in 95% ethanol	California, San Diego County, Marine Corps Base Camp Pendleton	A. Fisher	not recorded	2037	failed	no data
10	*Branchinecta lindahli*	29-Dec-12	fixed and preserved in 95% ethanol	California, San Diego County, Marine Corps Base Camp Pendleton	A. Fisher	not recorded	2038	failed	no data
11	*Branchinecta lindahli*	28-Dec-11	fixed and preserved in 95% ethanol	California, San Diego County, San Diego, Carmel Mountain Preserve	J. Snapp-Cook, et al.	egg sac only	1992	*beautiful sequence	6.62
12	*Branchinecta lindahli*	02-Apr-12	fixed and preserved in 95% ethanol	California, San Diego County, San Diego, Carmel Mountain Preserve	J. Snapp-Cook	1 gravid female	2026	*beautiful sequence	13
13	*Branchinecta lindahli*	02-Apr-12	preserved in 95% ethanol	California, San Diego County, San Diego, Carmel Mountain Preserve	J. Snapp-Cook	1 gravid female	2027	*beautiful sequence	11.3
14	*Branchinecta lindahli*	02-Apr-12	preserved in 95% ethanol	California, San Diego County, San Diego, Carmel Mountain Preserve	J. Snapp-Cook	1 gravid female	2034	failed	0
15	*Branchinecta lindahli*	02-Apr-12	preserved in 95% ethanol	California, San Diego County, San Diego, Carmel Mountain Preserve	J. Snapp-Cook	1 squished male	2028	*beautiful sequence	29
16	*Branchinecta longiantenna*	23-Mar-10	preserved in 70% ethanol	California, San Luis Obispo County, California Valley	Chris Powers	posterior half of single broken specimen	2005	failed	39.6
17	*Branchinecta lynchi*	27-Feb-01	fixed and preserved in 95% ethanol	California, San Luis Obispo County, Paso Robles	M. Dallas	1 specimen, not gravid, not obviously male	2032	contaminated; blasts as cladoceran	18.7
18	*Branchinecta lynchi*	13-Jan-04	fixed and preserved in 95% ethanol	California, Santa Barbara Co., Los Padres National Forest	T. Murphey	squished gravid female	2030	failed	0.3
19	*Branchinecta lynchi*	03-Feb-05	fixed and preserved in 95% ethanol	California, San Luis Obispo County	D. Hacker	posterior half of gravid female	2033	failed	51.4
20	*Branchinecta lynchi*	17-Feb-05	fixed and preserved in 95% ethanol	California, Santa Barbara Co., Los Padres National Forest, Branch Mountain Quad	T. Murphey	squished gravid female; all animals in this lot are pretty mangled	2031	failed	17
21	*Branchinecta mackini*	unknown	70% ethanol	Washington, Grant County	unknown	1 specimen	1991	failed	0.254
22	*Branchinecta mackini*	03-Apr-93	70% ethanol	California, San Bernadino County, Mojave Desert	C. Cash-Clark, T. Clark	1 specimen	2019	failed	0.16
23	*Branchinecta orientalis*	22-Aug-02	95% ethanol	Mongolia, Dundgovi’ aimag, near Sangiyn Dalay (Erdenedalay)	R. Wetzer, S.L. Boyce, N.D. Pentcheff	1 whole small specimen	2004	failed	24.4
24	*Branchinecta sandiegonensis*	09-Mar-05	preserved in 70% denatured ethanol, transferred to 70% ethanol	Mexico, Baja California, Tijuana, Jesus Maria Mesa	K.B. Clark	1 gravid female	2024	failed	0.7
25	*Branchinecta sandiegonensis*	13-Jan-11	preserved in 70% denatured ethyl alcohol, transferred to 70% ethanol	California, San Diego County, Brown Field Municipal Airport	D. Wolff	posterior half of gravid female	2029	failed	0
26	*Branchinecta sandiegonensis*	24-Nov-08	preserved in 95% ethanol	California, San Diego County, Ramona Water District, Ramona Spray Fields	E. Ervin	eggsac + furca from female	2023	failed	4.7
27	*Branchinecta sandiegonensis*	28-Dec-11	preserved in 95% ethanol	California, San Diego County, San Diego, Carmel Mountain Preserve	J. Snapp-Cook, et al.	anterior portion of female specimen	1995	failed	25.7
28	*Branchinecta sandiegonensis*	17-Dec-07	transferred to 95% ethanol Feb. 2011	California, San Diego County, Otay Mesa, Dexstar Property	C. Powers	1 male	2025	failed	49.2
29	*Branchinecta*	28-Dec-11	preserved in 95% ethanol	California, San Diego County, San Diego, Carmel Mountain Preserve	J. Snapp-Cook, et al.	1 specimen	1993	failed	7.5
30	*Branchipodopsis affinis*	22-Aug-02	95% ethanol	Mongolia, Dundgovi’ aimag, near Sangiyn Dalay (Erdenedalay)	R. Wetzer, S.L. Boyce, N.D. Pentcheff	1 small specimen	2001	failed	18.8
31	*Branchipodopsis affinis*	22-Aug-02	95% ethanol	Mongolia, Dundgovi’ aimag, near Sangiyn Dalay (Erdenedalay)	R. Wetzer, S.L. Boyce, N.D. Pentcheff	1 small specimen	2002	failed	49
32	*Chirocephalus*	22-Aug-02	95% ethanol	Mongolia, Dundgovi’ aimag, near Sangiyn Dalay (Erdenedalay)	R. Wetzer, S.L. Boyce, N.D. Pentcheff	1 whole squished animal	2018	*beautiful sequence	57.9
33	*Eubranchipus holmanii*	07-May-40	70% ethanol	Canada, Nova Scotia, Edinberg [sic]	D. Belk	1 male specimen	2015	contaminated; blasts as *Homo*	0
34	*Eubranchipus*	01-Apr-32	70% ethanol	Canada, Ontario, Saint Thomas	M.S. Ferguson	anterior end of broken specimen	2014	failed	0
35	*Eubranchipus*	30-Apr-99	70% ethanol	Minnesota, Bloomington	A.B. Forbes	1 female – doesn’t look well preserved	2022	failed	0
36	*Eubranchipus*	15-May-12	fixed and preserved in 95% ethanol	California, Lassen County, Poison Lake	M. Hauser, D. Striley	posterior half of the single mushy specimen	2020	*beautiful sequence	27.3
37	*Linderiella occidentalis*	19-Feb-92	70% ethanol	California, Tehama County, Tuscan Buttes	King, Mazzucco, Scuderi	2 pieces broken specimen	2000	contaminated; blasts as *Homo*	0.14
38	*Linderiella occidentalis*	24-Mar-92	fixative unknown – transferred to 70% ethanol	California, Tehama County, Dale’s Plains, Dale’s Lake	King, Gluesenkamp, Kloock	1 whole specimen	1987	failed	0.225
39	*Linderiella santarosae*	26-Mar-04	70% ethanol	California, Riverside County, Murrieta, Mesa de Colorado, Santa Rosa Plateau	M. Angelos	1 small female specimen	1999	contaminated; blasts as *Homo*	0.293
40	not identified	08-Jun-11	fixed and preserved in 95% ethanol	Utah, Wallsburg, near Provo-Jordan River Pkwy	M. Hauser	1 female specimen	2021	failed	47.6
40	*Phallocryptus*	22-Aug-02	95% ethanol	Mongolia, Dundgovi’ aimag, northwest of Delgerhangay (Khashaat/Delger Khanay Uul)	R. Wetzer, S.L. Boyce, N.D. Pentcheff	posterior half of adult specimen	2009	*beautiful sequence	10.3
42	*Pristicephalus comptus*	13-Apr-36	70% ethanol	Tennessee, Reelfoot Lake	unknown	1 specimen, this lot had previously dried and had been realcoholed	2012	failed	0
43	*Streptocephalus sealli*	15-Aug-55	70% ethanol	California, Tulare County, Yosemite, Tioga Pass	unknown	1 specimen, these had been previously dried and realcoholed	1998	failed	0.213
44	*Streptocephalus sealli*	15-Aug-55	95% ethanol	California, Mariposa County, Yosemite, May Lake Trail	unknown	posterior end of animal	1989	failed	0.18
45	*Streptocephalus texanus*	27-Aug-56	70% ethanol	New Mexico, Cain Ranch	S.F. Wood	dissected egg sack	2010	failed	0
46	*Streptocephalus woottoni*	30-Mar-06	70% ethanol	California, San Diego County, Camp Pendleton, Marine Corps Base	S. Baldwin	~5 phyllopods dissected off single specimen (only 1 specimen in the lot)	1994	failed	7.28
47	*Streptocephalus woottoni*	01-Apr-05	not recorded	California, San Diego County, Carlsbad, Poinsettia Lane Commuter Station Vernal Pools	J. Snapp-Cook	posterior half of male (already broken)	2016	*good sequence	16
48	*Streptocephalus woottoni*	29-Jan-03	preserved in 70% ethanol	California, Riverside County, Temecula	unknown	3-4 phyllopods removed from single specimen	1997	failed	6.8
49	*Tanymastix stagnalis*	12-Aug-34	70% ethanol	Denmark, Raabjerg Mile	E.W. Kaiser	3 broken pieces used	1996	failed	0.224
50	*Thamnocephalus platyurus*	01-Aug-56	70% ethanol	New Mexico, Gran Quivira	S.F. Wood	posterior portion	2011	failed	0

### DNA extractions

The starting material for DNA extractions varied among samples, one thoracopod to an entire animal, depending on total animal body size. Tissue samples were placed on paper towel to dry. Precipitation Reagent (Epicentre MMP03750) was added to each sample and vortexed vigorously for 10 sec., then centrifuged at 4 °C for 10 min. at 14,000 rpm. The supernatant (~300 µl) was transferred to a 2 ml tube. Genomic DNA was extracted and purified with a Quick-gDNA™ MiniPrep Kit (Zymo Research) following the manufacturer’s instructions, and eluted in a final volume of 60 µl of distilled water (in two elutions of 30 µl). Double-stranded DNA concentration of extractions was quantified using a Qubit 1.0 Fluorometer (Life Technologies) (see Table 1).

### PCR protocols

The mt16SrDNA fragment was amplified with universal 16Sar and 16Sbr primers (Palumbi et al. 1991) and both strands were sequenced. PCR reactions were done in a final volume of 50 µl. The volume of DNA used in each reaction varied from 2–25 µl depending on the DNA concentration measured on the Qubit. When possible, we tried to use at least 50 ng of DNA. Two different PCR reaction setups were used, as some samples successfully amplified with one, but not with the other. The first setup consisted of 10 µl of GoTaq Promega Buffer 5x, 5 µl of 2.5 mM MgCl_2_, 4 µl of a 10 mM dNTP mixture, 2 µl of each primer at 20 µM, and 0.3–0.5 µl of GoTaq Polymerase at 5 U/µl (Promega). The second setup consisted of 25 µl of a 2x PCR Master Mix with 1.5 mM MgCl_2_ (Thermo Scientific), and 1 µl of each primer at 20 µM. Both positive and negative controls were run in each experiment. Amplifications were performed in a BIO-RAD S1000 Thermal Cycler, with the following thermocycler conditions: an initial step of 5 min. at 95 °C, 35 cycles of 30 sec. at 95 °C, 30 sec. at 48 °C, 45 sec. at 72 °C, and a final extension of 10 min. at 72 °C. Amplifications were checked by running 5 µl of the PCR product on a 1.5% agarose gel. All failed amplifications were retried at least twice with different polymerases, buffers, and MgCl_2_ concentrations. Successful PCR reactions were then purified with a DNA Clean and Concentrator-5 Kit (Zymo Research) and sequenced with both primers at Laragen Inc, Culver City, CA. Chromatograms were visually inspected and edited with 4Peaks (Griekspoor and Groothuis 2014).

### Contamination screening

Sequences were edited and contigs assembled in the software program Sequencher (Gene Codes Corporation 2004), and all contigs were BLAST searched in the NCBI database to verify they were not contaminants (*i.e.*, that sequence was indeed from the taxon of interest).

### Statistical testing

A Fisher’s exact test (two-tailed, α=0.05) was used to determine whether there was a statistically significant difference in sequencing success between the ethanol-preserved and other samples (Zar 1999). A Qubit 1.0 Fluorometer (Life Technologies) was used to quantify double-stranded DNA (Table 1). A one-tailed Mann-Whitney U test (Zar 1999) was used to assess statistical significance between double-stranded DNA concentration and amplification success.

## Results

Of the 50 individual anostracan samples on which we attempted PCR amplification, 13 were known to have been fixed and preserved in pure 95% ethanol, and 37 samples had unknown preservation histories but were suspected of being fixed and stored in denatured ethanol sometimes for years, until they were incorporated into the LACM collection. Of the samples fixed and preserved in 95% ethanol, 62% (8 out of 13) yielded useable mt16SrDNA sequences. In contrast, of the samples with unknown fixative and preservative history, only 3% (1 out of 37) yielded useable mt16SrDNA. The nine sequences generated here are available on GenBank (see Table 2). Sequencing success between samples fixed and preserved in ethanol and other samples was significantly different (Fisher’s exact test, two-tailed, P < 0.0007).

**Table 2.** Nine new mt16SrDNA Anostraca sequences: taxonomy, Genbank number, and locality information. All specimens and DNA are deposited in the collections of the Natural History Museum of Los Angeles County. Required permits are on file at USFWS and/or LACM.

Genus/species	Genbank No.	Locality
Artemiidae: *Artemia monica*	KF790567	USA, California, Mono County, Mono Lake, ~38.011°N ~119.012°W, hypersaline lake, 95% ethanol. 1 Jan 2010. Coll. M. Hauser. RW12.244.2008
Branchinectidae: *Branchinecta lindahli*	KF790568	USA, California, San Diego County, San Diego, Carmel Mountain Preserve, 32.929°N, 117.22°W, vernal pool 4 in. deep, 8 ft. wide, 28 ft. long, water slightly murky, 63 µm net, 95% ethanol. 28 Dec 2011. JS pool #21, MBPC 11637. Coll. J. Snapp-Cook, C. Lieberman, A. Wall, P. Sun, R. Wetzer. RW13.042.1992
Branchinectidae: *Branchinecta lindahli*	KF790569	USA, California, San Diego County, San Diego, Carmel Mountain Preserve, 32.933°N, 117.215°W, vernal pool in dirt road, 95% ethanol. 2 Apr 2012. City ID # 22, js_fs_37, MBPC13258. Coll. J. Snapp-Cook. RW13.047.2026
Branchinectidae: *Branchinecta lindahli*	KF790570	USA, California, San Diego County, San Diego, Carmel Mountain Preserve, 32.932°N, 117.215°W, vernal pool in dirt road, 95% ethanol. 2 Apr 2012. City ID # 20, js_fs_38, MBPC13259. Coll. J. Snapp-Cook. RW13.048.2027
Branchinectidae: *Branchinecta lindahli*	KF790571	USA, California, San Diego County, San Diego, Carmel Mountain Preserve, 32.928°N, 117.22°W, vernal pool in dirt road, 95% ethanol. 2 Apr 2012. City ID # 26, js_fs_35, MPBC13256. Coll. J. Snapp-Cook. RW13.046.2028
Chirocephalidae: *Chirocephalus* sp.	KF790572	Mongolia, Dundgovi’ aimag, near Sangiyn Dalay (Erdenedalay), 46.135°N, 105.106°E, 2 acre pond, 0-1 ppt, 23.2°C, 63 µm mesh net, 95% ethanol. 22 Aug 2002. GPS#016, Mongolia Expedition 2002, MBPC 431. Coll. R. Wetzer, S.L. Boyce, N.D. Pentcheff. RW13.034.2018
Chirocephalidae: *Eubranchipus* sp.	KF790573	USA, California, Lassen County, Poison Lake, 40.659°N, 121.197°W, temporary lake, hand, 95% ethanol. 15 May 2012. Coll. M. Hauser and D. Striley. RW12.242.2020
Streptocephalidae: *Streptocephalus woottoni*	KF790574	USA, California, San Diego County, Carlsbad, Poinsettia Lane Commuter Station Vernal Pools, large pool at southern end of complex, 33.108°N, 117.318°W, vernal pool 15 m x 30 m, 12-24 inches deep, murky water, 1 Apr 2005. MBPC 10061. Coll. J. Snapp-Cook. RW13.007.2016
Thamnocephalidae: *Phallocryptus* sp.	KF790575	Mongolia, Dundgovi’ aimag, northwest of Delgerhangay (Khashaat/Delger Khanay Uul), 45.424°N, 104.481°E, large lake reduced to tiny watering hole, 11 ppt, 28°C, 63 µm mesh net, 95% ethanol. 22 Aug 2002. GPS#020, Mongolia Expedition 2002, MBPC 435. Coll. R. Wetzer, S. L. Boyce, N. D. Pentcheff. RW13.036.2009

The one-tailed Mann-Whitney U Test showed that there was a difference (at the α = 0.05 level) between Qubit measurements of double-stranded DNA concentration for successful sequences vs. failed sequences, when amplifications of contaminants were considered as failed amplifications. However, direct examination of the data (see Table 1) showed that DNA concentration was a very poor predictor of sequencing success (except for the case of 0 or near-0 readings, which invariably failed).

## Discussion

### Existing museum specimens

Specimens known to be collected and preserved in 95% ethanol were successfully extracted, amplified and sequenced at a much higher success rate than those with unknown preservation history (probably denatured alcohol). Although some specimens enumerated in Table 1 indicate that they were preserved in 95% ethanol, label data does not distinguish denatured from not denatured ethanol, and the additional collector information provides only hints of the actual preservative in most cases. Specimens preserved in 70% denatured ethanol in the field and subsequently transferred to 95% not denatured ethanol failed. Based on previous experimentation, neither acetone nor isopropyl alcohol preservation resulted in successful amplification, so these preservatives were excluded from this analysis. Similarly, specimens known to have been exposed to formalin were excluded, as all previous attempts have failed for these types of broad taxonomic, spatial, and temporal studies using Sanger sequencing approaches (RW, pers. obs.). The interactions of formalin with specimens result in denaturation of the DNA and a variety of other reactions (Tang 2006). Additionally, over time, oxidation of formaldehyde in formalin to formic acid produces an acidic solution resulting in the scission of DNA. The smaller the specimen, the greater the effect, and the lower the likelihood of success of long strand DNA extraction. The Tang (2006) study, commissioned by the National Academy of Sciences, provides a detailed (and discouraging) review of DNA extraction and sequencing from formalin-fixed biological samples.

### Collecting recommendations

Our aim was to maximize the scientific value of specimens and their biological usefulness for future studies. First, the results of our study make a very compelling case that initial specimen fixation and preservation in the field should use 95% ethanol — *not* denatured ethanol or other alcohols. If not denatured ethanol is unavailable, we recommend fixation and preservation in 100 proof (or higher) vodka, rum, Everclear™, or similar drinking alcohol, rather than any sort of denatured alcohol. This method, although the next best choice, has been successfully used during expeditionary work by one of us (RW) since the mid-1980s. Although 100 proof spirits are only 50% ethanol by volume, the quality of the alcohol matters more than the concentration — if you cannot drink it, it’s *not* good for specimens. Second, specimens should always be in a volume ratio of at least 3:1 alcohol:specimens to avoid degradation from dilution of preservative by body fluids. Third, once specimens are returned from the field, ethanol should be replaced with fresh 95% not denatured ethanol to compensate for dilution of the preservative by water extracted from specimen tissue.

In addition to the changes we suggest for the fixation and preservation, we also suggest changes to the type and number of voucher specimens being deposited after an environmental impact report is completed. We recommend accessioning specimens of all species, whether listed or not (*e.g.* whether endangered or threatened, or not). For example, simply accessioning both the listed and non-listed species will make it possible to definitively address questions about hybridization between *Branchinecta sandiegonensis* and *Branchinecta lindahli*. Furthermore, depositing all specimens collected for a survey, not just a single voucher specimen for each species, will increase sample sizes to enable population level molecular studies.

These small improvements to collecting protocols will make it possible to derive high-quality data for future biodiversity and phylogeographic research. Since the sacrifice of endangered and non-endangered crustaceans is necessary to evaluate their presence and abundance in the wild, they can become a valuable historic resource if properly curated and deposited.
